# Clinical features and outcome in 25 dogs with respiratory‐associated pulmonary hypertension treated with sildenafil

**DOI:** 10.1111/jvim.15679

**Published:** 2019-12-09

**Authors:** Lynelle R. Johnson, Joshua A. Stern

**Affiliations:** ^1^ Department of Medicine and Epidemiology University of California, Davis Davis California

**Keywords:** chronic bronchitis, interstitial lung disease, tracheobronchomalacia

## Abstract

**Background:**

Pulmonary hypertension (PH) can develop secondary to many common cardiopulmonary diseases, and the use of sildenafil has improved care of affected dogs.

**Objective:**

To evaluate response to sildenafil in dogs with respiratory‐associated PH.

**Animals:**

Twenty‐five dogs with PH.

**Methods:**

Prospective clinical trial. Doppler echocardiography identified dogs with moderate to severe PH, and additional tests were performed to detect underlying diseases. A 17‐point quality of life (QOL) questionnaire was completed, and sildenafil was prescribed, along with other medications deemed necessary for the management of clinically diagnosed respiratory diseases. After 30 days, dogs returned to the hospital for repeat echocardiogram and QOL survey.

**Results:**

The median age was 12.4 years, and most dogs were small breed dogs (median weight, 6.5 kg). Syncope (64%), cough (56%), and respiratory difficulty (32%) were the most common presenting complaints. Respiratory diseases associated with PH included tracheobronchomalacia, pulmonary fibrosis, inflammatory airway disease, and brachycephalic syndrome, with multiple diseases in some dogs. Eight of 25 dogs (32%) died or were euthanized within 1 month. In the remaining dogs, tricuspid regurgitation pressure gradient (83.0 ± 17.4 mm Hg before, 55.4 ± 17.4 mm Hg after) and QOL scores were significantly improved after 1 month of sildenafil. Fifty percent mortality was reached 6 months after study entry, with 4 dogs alive 5 years after diagnosis.

**Conclusions and Clinical Importance:**

Sildenafil responsiveness is variable in dogs with respiratory‐associated PH, but improved QOL was demonstrated in dogs surviving >1 month, and long‐term survival was noted in some cases.

**Abbreviations**
BOAS: brachycephalic obstructive airway syndrome
MMVD: myxomatous mitral valve disease
PA: pulmonary artery
PAP: pulmonary artery pressure
PH: pulmonary hypertension
QOL: quality of life
TBM: tracheobronchomalacia
TR: tricuspid regurgitation
TRPG: tricuspid regurgitation pressure gradient

## 1 INTRODUCTION

Pulmonary hypertension (PH) is characterized by a persistent and pathologic increase in pulmonary artery (PA) systolic and mean pressure exceeding 30 and 20 mm Hg, respectively.^1^ Originally, PH was characterized as either a primary disease of the vasculature or as a secondary vascular disorder that developed in association with various cardiopulmonary diseases. The World Health Organization has identified 5 classes of diseases that can result in PH. In the recent classification,^2^ Group 1 (pulmonary arterial hypertension) was ascribed to primary diseases of the vasculature, including idiopathic, familial, toxin‐induced, congenital heart disease, and veno‐occlusive disease, among others. Group 2 (pulmonary venous hypertension) was related to left‐sided heart disease and chronic increases in left atrial pressure. Group 3 PH occurred with lung diseases or hypoxemia, including obstructive pulmonary disease, interstitial lung disease, alveolar hypoventilation, and sleep apnea. Group 4 comprised chronic thromboembolic PH, and group 5 included systemic and other disorders. Precise mechanistic explanations for the development of vascular remodeling in these disorders remain unclear,^2^ with imbalance of endothelium‐derived mediators, intermittent or sustained hypoxia‐induced proliferation and inflammation, and epigenetic factors believed to play roles.^3^, ^4^, ^5^, ^6^, ^7^, ^8^

In veterinary medicine, group 1 disorders (pulmonary arteriopathy, capillary hemangiomatosis, and veno‐occlusive disease) are rare and exclusively identified at necropsy,^9^, ^10^, ^11^ although some of these disorders might be suspected clinically. The second classification includes animals with PH associated with left‐sided heart disease, which is often the most commonly identified cause in dogs,^12^, ^13^, ^14^, ^15^ although the group of animals under investigation is likely important in determining the commonality of the underlying disease process associated with PH. Groups 3 through 5 also have been commonly described in veterinary studies of PH,^12^, ^13^ although definitive diagnosis of the underlying disease process related to PH for dogs in these groups is challenging because of the need for invasive testing including histopathology to confirm many of these disease processes. For example, pulmonary thromboembolism remains difficult to diagnose antemortem and can be found in dogs with normal d‐dimer concentrations^16^; tracheobronchomalacia (TBM) can require radiographic, fluoroscopic, and bronchoscopic assessment^17^; and, pulmonary fibrosis requires histopathologic documentation,^18^ although high‐resolution computed tomography increasingly is used in the diagnosis.^19^ Clinically, PH has been reported in a number of these diseases with diagnosis based on clinical suspicion, signalment, physical examination findings, and diagnostic imaging.^12^, ^13^, ^20^ However, small numbers of dogs are generally available for inclusion in studies of clinical diseases in veterinary medicine, and diagnostic evaluation is limited by owner finances and consent. Dogs can suffer from multiple concomitant respiratory disorders as well as cardiac disease, further confusing categorization of specific disease processes that could result in PH. Finally, a miscellaneous category (group 5) including dogs with neoplasia, endocrinopathies, systemic inflammatory disease, and dogs with no obvious disease associated with PH has comprised 4%‐38% of affected animals in various studies,^12^, ^13^, ^21^ further complicating evaluation of the underlying diseases in dogs with PH.

Despite challenges in identifying the underlying disease processes associated with PH, the syndrome has been identified with increasing frequency because of advances in Doppler echocardiography, which provides a minimally invasive estimation of pulmonary artery pressure (PAP) by evaluation of the velocity of the tricuspid regurgitant jet.^12^, ^13^, ^14^, ^15^, ^20^, ^21^, ^22^, ^23^, ^24^ Echocardiographic measures are impacted by operator and patient characteristics,^22^ and it is important to remember that Doppler can provide only an approximation of PAP. Estimated PAP showed a significant and moderate correlation with results obtained during right heart catheterization in healthy Beagles, but variation increased at higher pressures.^23^ It is likely that these variables also play a role in dogs with clinically‐observed PH, although comparisons of Doppler echocardiography to more invasive methodology have not been performed in clinical patients to date.

Sildenafil is recommended for use only in group 1 human patients but it has been evaluated in both retrospective and prospective studies of dogs with PH associated with different cardiopulmonary diseases, with variable results reported.^21^, ^22^, ^24^ Our prospective study sought to compare response to a standard 1‐month regimen of sildenafil on the tricuspid regurgitation pressure gradient (TRPG) and quality of life (QOL) score in dogs with respiratory‐associated causes of moderate to severe PH. In addition, we sought to refine our understanding of survival in dogs with PH related to respiratory causes.

## 2 MATERIALS AND METHODS

This study was approved by the Institutional Animal Care and Use Committee at the University of California‐Davis. Dogs with clinical signs and physical examination findings consistent with PH associated with respiratory disease or dysfunction as defined below were prospectively identified by attending clinicians at the William R. Pritchard Veterinary Medical Teaching Hospital between 2014 and 2016. Inclusion criteria required the presence of a minimum estimated right ventricular systolic pressure gradient of 50 mm Hg^12^ (deemed moderate PH) in the absence of outflow tract obstruction or any observed congenital valvular heart defects identified by echocardiography. Exclusion criteria included a left atrial: aortic size >1.6^25^ as measured in the Evaluation of Pimobendan In dogs with Cardiomegaly trial or diagnosis of congenital heart disease. Dogs with acute congestive heart failure or evidence of ruptured chordae tendinae also were excluded from the study.

Two‐dimensional, M‐mode, color, and spectral Doppler echocardiography (Philips IE33, Philips Healthcare, Andover, Massachusetts) was performed by a board‐certified cardiologist or resident in training, with direct supervision by a board‐certified cardiologist. All raw echocardiographic data were obtained and saved for offline analysis at a digital workstation (Syngo Dynamic Workplace, Version 10.0.01_HF04_Rev5 [Build 2884], Siemens Medical Solutions, USA, Inc, Malvern, Pennsylvania). Dogs were not sedated and were manually restrained in right and left lateral recumbency. Standard imaging techniques were used with continuous ECG monitoring.^26^ Studies were not routinely repeated after injection of agitated saline unless the attending cardiologist had a high suspicion for congenital heart disease based on physical examination, echocardiographic or historical findings.

Tricuspid regurgitation (TR) was identified by color Doppler and the velocity obtained using continuous wave spectral Doppler. Special attention was given to selecting the imaging window that provided the most parallel alignment between the spectral Doppler cursor and tricuspid regurgitant jet. A modal tricuspid regurgitant velocity measurement >3.5 m/sec was required for inclusion in the study and was transformed to an estimated pressure gradient using the modified Bernoulli equation without adding any estimate of right atrial pressure. All echocardiographic measurements were obtained by a board‐certified cardiologist or resident in training and verified by the board‐certified cardiologist. Peak TR jet velocity was measured as the modal velocity of complete flow profiles and measured only when the patient was in sinus rhythm.

Respiratory diagnoses in dogs with PH were determined based on history, signalment, physical examination, clinicopathologic findings, and radiographic changes. Additional diagnostic tests were performed (eg, fluoroscopy, bronchoscopy with bronchoalveolar lavage) at the discretion of the attending clinician and with owner consent after echocardiographic recognition of PH. Specific respiratory diseases were finalized by one of the investigators (L.R.J.) after review of records and images. Diseases included TBM, characterized by dynamic change in airway diameter evident on digital radiography, fluoroscopy, or bronchoscopy; inflammatory airway disease (chronic bronchitis or eosinophilic lung disease) based on radiographic findings of a bronchial infiltrate, airway cytology, or positive response to corticosteroids; infectious respiratory disease, as determined by bronchointerstitial or alveolar radiographic infiltrates, airway cytology and culture, or positive response to antibiotics; and, interstitial lung disease, suspected based on breed, and the presence of tachypnea, auscultation of crackles, and a diffuse interstitial pattern on radiographs with or without documentation of hypoxemia. Brachycephalic obstructive airway syndrome (BOAS) was diagnosed based on breed and facial conformation.

At entry into the study, owners completed a previously validated 17‐point QOL questionnaire,^27^ which assessed the presence of clinical signs impacting breathing and functional status and assigned severity to the level of debility on a scale of 0 (not at all) to 5 (very much). This survey was developed based on widely accepted clinical signs of cardiac disease, which closely match those found with respiratory diseases. No modifications were made to the survey, and all questions were answered by owners, including whether increased urination accidents or vomiting were documented. The sum of the 17‐point survey ranged from 0 to 85, with higher scores reflecting poorer QOL.

After diagnostic testing, sildenafil was prescribed at a target dosage of 2‐4 mg/kg/day, with dosing adjusted as needed to accommodate available pill sizes. Owners were asked to observe dogs for hypotensive adverse effects potentially related to sildenafil such as weakness or lethargy after administration. The study protocol requested limited use of additional medications, but dogs were treated with antibiotics when infection was suspected or documented, corticosteroids when inflammatory or eosinophilic airway disease was diagnosed, and extended‐release theophylline for potential assistance in management of TBM. After 1 month of sildenafil treatment, dogs returned to the hospital for repeat physical examination, cardiovascular and echocardiographic assessment, and completion of a post‐treatment QOL questionnaire. Pre‐ and post‐treatment questionnaires were submitted to the study manager, and results were not available to the primary clinician or the echocardiographer at the time of reevaluation.

Age, breed, weight, and sex were retrieved from the medical record. Presenting complaints and duration of clinical signs were retrieved for each dog. Survival was determined by contact with referring veterinarians and owners and was defined as the number of months beyond diagnosis to death or euthanasia for clinical signs related to cardiopulmonary disease. Dogs still alive or those euthanized for reasons unrelated to PH at the time of follow‐up were right‐censored in the survival analysis.

### 2.1 Statistical analysis

Statistical analysis was performed using a commercially available statistics program (GraphPad Prism 5.0f, San Diego, California). Visual inspection and D'Agostino and Pearson omnibus normality test were used to assess the distribution of results for age, body weight, duration of signs, QOL scores (before, after, and change), estimated TRPG (before, after, and change), and survival data. Normally‐distributed data are presented as mean ± SD with inclusion of range when clinically relevant, and nonparametric data are presented as median with range. To account for missing data in dogs that died before 1‐month follow‐up, post‐treatment QOL score was assigned the maximal value of 85, and the Wilcoxon signed rank test for paired data was used to compare pretreatment and posttreatment scores. In dogs that completed the 1‐month treatment trial, QOL scores and TRPG pre‐ and post‐sildenafil were compared using a paired *t* test. Tricuspid regurgitation pressure gradient and QOL scores pre‐ and post‐sildenafil were compared between groups of dogs with and without a history of syncope. Correlation of TRPG with QOL score and survival was assessed using linear regression. Survival analysis was performed using a Kaplan‐Meier estimate. For all analyses, significance was set at *P* < .05.

## 3 RESULTS

Pulmonary hypertension associated with respiratory diseases was identified in 25 dogs. The group was comprised of 4 Chihuahuas, 4 West Highland White Terriers, 3 mix breed terrier type dogs, 2 Pekingese, 2 Shih Tzu, and 1 each of Affenpinscher, Cavalier King Charles Spaniel, Jack Russell Terrier, Maltese, Miniature Dachshund, Pomeranian, Poodle, Pug, Shetland Sheep dog, and Tibetan terrier. Twelve dogs were spayed females, 2 were intact males, and 11 were neutered male dogs. Median age was 12.4 years (range, 3.6‐15.7 years) and median weight was 6.5 kg (range, 2.2‐32 kg), with 7 dogs <5 kg, 13 dogs 5‐10 kg, 4 dogs 10‐14 kg, and 1 dog 32 kg. Syncope was the most common presenting complaint (16/25; 64%) with a duration of 1 day to 9 years (median, 2 months). Cough was reported in 14 of 25 dogs (56%) with duration ranging from 2 days to 3 years (mean, 14.8 ± 11.7 months; median, 12 months), and difficult breathing or respiratory distress was described in 8 of 25 dogs (32%) with duration ranging from 2 weeks to 2.5 years (median, 5.5 months). Quality of life score at study entry ranged from 5 to 79 (out of a maximum of 85) with a mean value of 37 ± 17.

Diagnosis of PH was based on detection of a tricuspid regurgitant jet in all 25 dogs, with pressure gradients ranging from 50 to 131 mm Hg (mean, 83 ± 21.4 mm Hg). Pulmonary hypertension was moderate (50‐74 mm Hg) in 11 dogs and severe (≥75 mm Hg) in 14 dogs. No correlation was found between TRPG and QOL score (*P* = .29) at study entry. Tricuspid regurgitation pressure gradient and QOL scores did not differ between the groups of dogs with and without a history of syncope (*P* = .20 and .34; Table 1).

**Table 1:** QOL scores and TRPG in dogs with and without syncope as a presenting complaint

	Dogs with syncope (n = 16)	Dogs without syncope (n = 9)	*P* value
QOL score before	40.7 ± 19.5	31.4 ± 9.2	.20
TRPG before (mm Hg)	79.8 ± 17.2	88.6 ± 27.6	.34
QOL score after	19.1 ± 9.5	15.8 ± 17.0	.63
TRPG after (mm Hg)	60.9 ± 19.9	48.0 ± 12.0	.24

Abbreviations: QOL, quality of life; TRPG, tricuspid regurgitation pressure gradient.

Multiple respiratory conditions were present in some dogs (Table 2). Diagnoses included TBM in 9 dogs based on a combination of history, physical examination findings, and documentation of changes in airway luminal diameter based on radiography (n = 6), fluoroscopy (n = 1), or bronchoscopy (n = 2). Interstitial lung disease was clinically suspected in 10 dogs (4 West Highland white terriers, 2 terrier mix dogs, and 1 each of the Affenpinscher, Shih Tzu, Pomeranian, and Tibetan Terrier) based on the radiographic evidence of diffuse interstitial infiltrates and labored breathing, with or without auscultable crackles. Arterial blood gas analysis was performed on 1 West Highland white terrier and identified a P_a_O_2_ of 36.6 mm Hg (normal, 80‐100 mm Hg). This sample was confirmed as arterial by comparison to P_v_O_2_. Inflammatory (n = 3) or infectious (n = 3) disease was diagnosed in 6 dogs by bronchoscopy (n = 2) or response to treatment with corticosteroids or antibiotics, respectively (n = 4). Brachycephalic syndrome was present in 5 dogs based on breed and physical examination findings, and 1 additional dog had stridor and airway obstruction ascribed to cervical tracheal collapse. Necropsy findings were available in 2 dogs in which a clinical diagnosis of pulmonary fibrosis had been made based on breed (terrier mix and Tibetan Terrier), crackles on pulmonary auscultation, and diffuse interstitial infiltrates on radiographs. One dog died 4 days after diagnosis and histopathology disclosed chronic occlusive pulmonary thromboemboli. One dog improved clinically and radiographically on sildenafil and antibiotics but was euthanized before posttreatment follow‐up, likely because of a poor appetite. Histopathology at necropsy was consistent with pulmonary capillary hemangiomatosis.

**Table 2:** Distribution and combinations of suspected respiratory causes of pulmonary hypertension in 22 dogs of this study

	Number of affected dogs
Interstitial lung disease	8^a^
Interstitial lung disease + BOAS	1
Interstitial lung disease + tracheobronchomalacia	1
Tracheobronchomalacia	4
Tracheobronchomalacia + BOAS + inflammation	1
Tracheobronchomalacia + BOAS	1
Tracheobronchomalacia + inflammation	1
Tracheobronchomalacia + infection	1
Infection	1
BOAS + infection	1
BOAS	1
Inflammation (eosinophilic)	1

*Note*: Three dogs were considered to have respiratory‐associated pulmonary hypertension but could not be assigned a specific etiology. These dogs had cough and syncope in association with normal left atrial size (n = 3), bronchointerstitial infiltrates (n = 2), and bronchiectasis (n = 1), and were treated with sildenafil alone.

Abbreviation: BOAS, brachycephalic obstructive airway syndrome.

a Two dogs in this category were ultimately diagnosed with pulmonary vascular disease on necropsy.

Eighteen dogs were discharged on sildenafil alone at dosages ranging from 1.75 to 6.82 mg/kg/day with a mean of 4.2 ± 1.1 mg/kg/day divided q8h or q12h, depending on the tablet size. Additional medications prescribed in 7 dogs included extended‐release theophylline (n = 4), antibiotics (n = 4), and corticosteroids (n = 3). Eight dogs died within 4 weeks of discharge, 7 of which were on sildenafil alone, with 1 dog on sildenafil and an antibiotic. Posttreatment evaluation was performed 28.7 ± 2.8 days after diagnosis in 15 dogs, 10 of which received sildenafil alone and 5 of which were treated with additional medications. Quality of life surveys were unavailable for 10 dogs because of euthanasia or death before follow‐up (n = 8) or because surveys were not completed or lost (n = 2). Quality of life scores at study entry were worse in dogs that did not survive to follow‐up (45.3 ± 17.3) in comparison to those that did (32.1 ± 15, *P* = .054). Tricuspid regurgitation velocity could not be assessed posttreatment in 9 dogs because of death or euthanasia (n = 8) and failure to return for follow‐up (n = 1). Results obtained for TRPG at study entry did not differ for dogs that did not survive to follow‐up (81.4 ± 19.9 mm Hg) in comparison to those that did (85.7 ± 24.8 mm Hg, *P* = .64).

**Figure 1:**
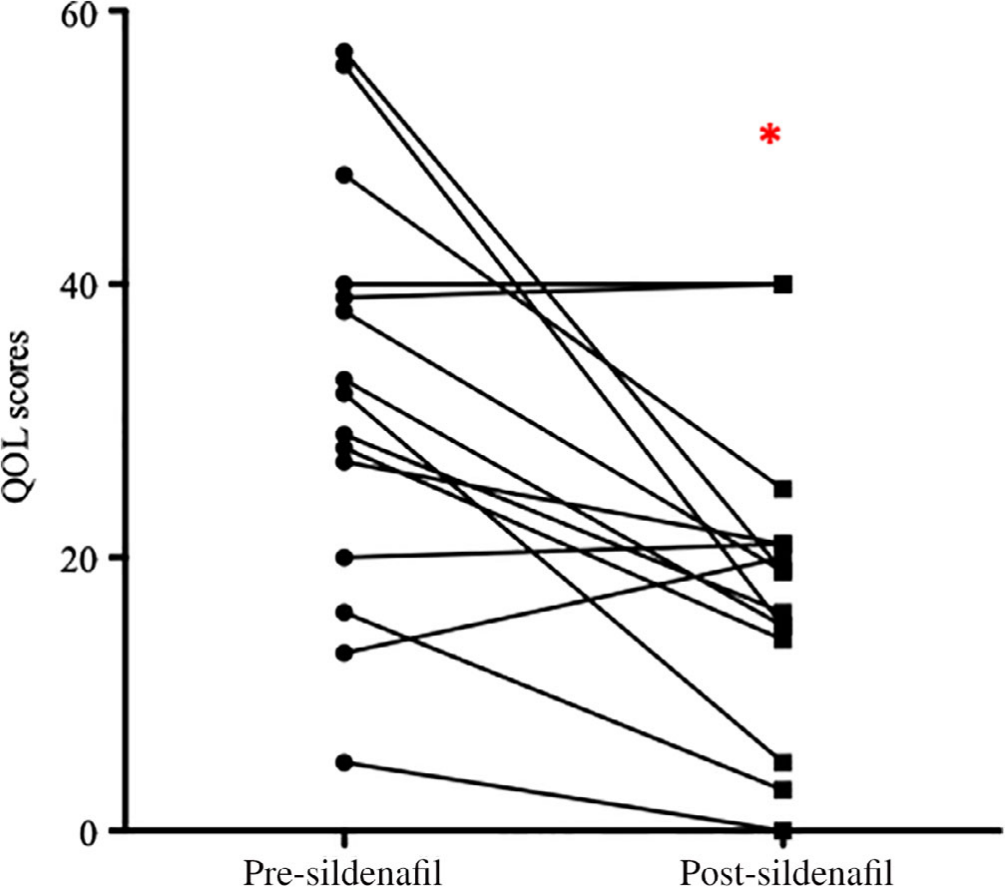
Quality of life (QOL) scores out of a maximum of 85 points before and after 1 month of treatment with sildenafil (n = 15). Although 4 dogs demonstrated a worsening in QOL as indicated by an increased score, overall values were significantly lower posttreatment, *P = .002

**Figure 2:**
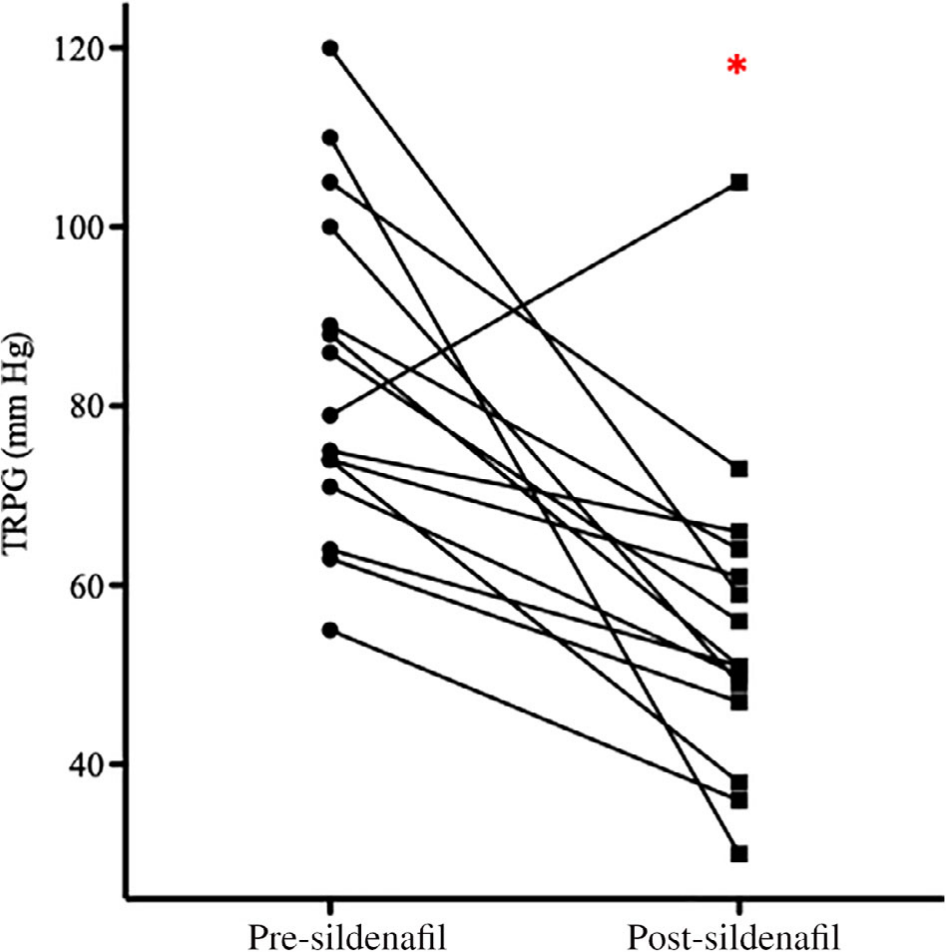
Change in tricuspid regurgitation pressure gradient (TRPG) in dogs (n = 16) following 1 month of sildenafil was significantly lower than at study entry (*P = .0006) although one dog had an increase in TRPG

In 8 of 25 dogs (32%) that died before completing the month of treatment, a maximal QOL score (85) was assigned as a follow‐up result, and statistical analysis identified no difference in before and after results (*P* = .73). Quality of life score after 4 weeks of sildenafil treatment in 15 dogs completing 1 month of treatment was significantly improved (18.2 ± 11.3), with a mean change of 14 ± 14 points (*P* = .002, Figure 1). No difference was found in response between dogs treated with sildenafil alone and those that were prescribed additional medications (*P* = .24). Four dogs had no change or worsening in their QOL score, and suspected respiratory etiologies in those dogs included TBM, BOAS, TBM and BOAS, and BOAS with infection. Despite worsened QOL scores, these dogs survived 2‐9.5 months after diagnosis, with 3 dogs being managed using sildenafil alone. Tricuspid regurgitation pressure gradient measurements after 4 weeks of sildenafil treatment in 16 dogs available for follow‐up were significantly lower than pretreatment (*P* = .0006) with a mean decrease of 24 ± 21 mm Hg (range, −26 to 61 mm Hg; Figure 2). No difference was found in TRPG response between dogs treated with sildenafil alone and those that received additional medications (*P* = .28). Improvement in QOL score did not correlate with the change in TRPG (*P* = .18).

**Figure 3:**
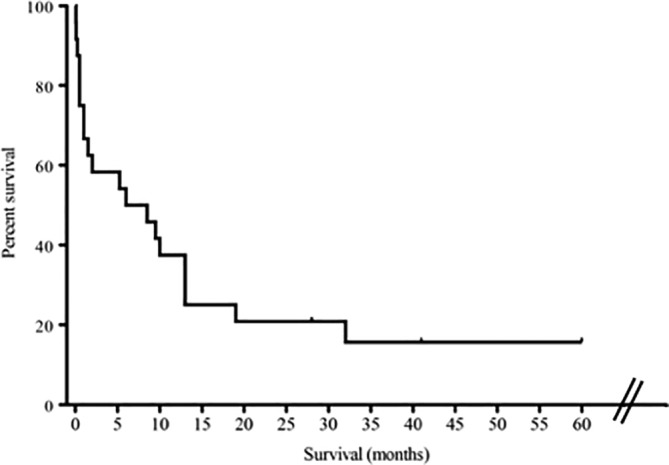
Kaplan‐Meier survival curve revealed a 50% survival of 6 months for all dogs (n = 25) with respiratory‐associated pulmonary hypertension

**Figure 4:**
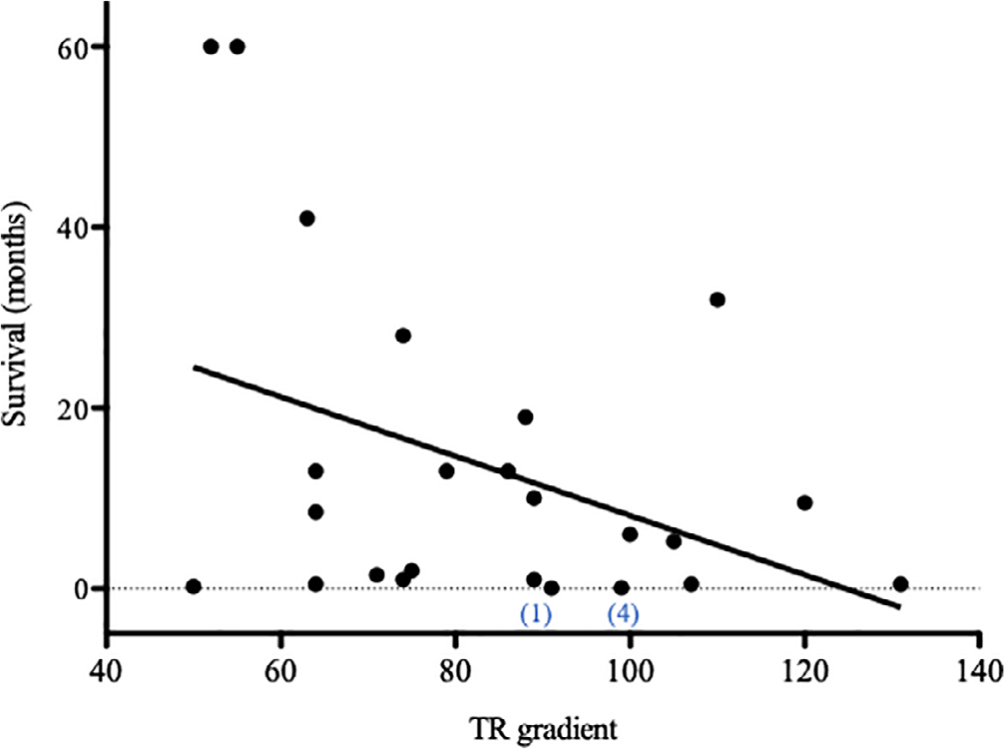
Tricuspid regurgitation pressure gradient measured at study entry in 25 dogs demonstrated a weak negative correlation with survival, *P* = .05. Numbers in brackets reflect confirmed classification of 2 dogs based on necropsy findings of pulmonary capillary hemangiomatosis (1) and chronic thromboembolism (4). Necropsies were unavailable for any other dog in the study

Owners reported no adverse effects of weakness or lethargy potentially related to sildenafil. Ten dogs died with signs related to cardiopulmonary disease (eg, cough, respiratory difficulty, syncope) 3 days to 32 months after diagnosis (median, 3.4 months). Two dogs were euthanized 1 week and 6 months after diagnosis of PH for renal failure and retrobulbar disease, respectively. Euthanasia was performed in 8 dogs because of signs related to cardiopulmonary disease (eg, cough, respiratory distress, syncope) 2 days to 19 months after diagnosis (mean, 6.7 ± 7.0 months). One dog was lost to follow‐up after 28 months on sildenafil and 4 dogs were known to be alive 28 to 60 months post‐diagnosis. No difference was found in survival between 9 dogs treated with sildenafil alone (median, 10 months; range, 1.5‐41 months) and 5 dogs treated with sildenafil in combination with other drugs (n = 5; median 19 months; range, 9.5‐60 months; *P* = .11), although our study could have been underpowered to detect a difference. The dog with the longest survival (>5 years) presented with syncope because of PH and cough associated with eosinophilic bronchitis. This dog experienced resolution of all clinical signs in response to sildenafil and glucocorticoids and ultimately was taken off medications. Fifty percent mortality for all dogs in this study was reached at 6 months (Figure 3), whereas in dogs that completed 1 month of sildenafil treatment, 50% mortality approached 1 year. A weak negative relationship was found between TRPG at study entry and survival (*P* = .05; R^2^ = .16; Figure 4).

## 4 DISCUSSION

Our prospective study confirmed the efficacy of sildenafil in improving QOL and also in decreasing TRPG in most dogs with PH associated with a respiratory disorder. It also suggested that the severity of TRPG at the time of diagnosis plays a role in prognosis for dogs with respiratory‐associated PH. This finding is clinically important because Doppler echocardiography is susceptible to a number of potential errors in estimating PA pressure and thus might be considered of questionable diagnostic, therapeutic, or prognostic value. However, although limitations of Doppler echocardiography in the diagnosis of PH must be considered, it remains an important clinical tool because right heart catheterization, which is the gold standard for documentation of PH, has not been used in clinical veterinary studies to date.

The original report examining the effects of sildenafil on PH in dogs with cardiac and respiratory origins of PH reported a median decrease in estimated PA pressure of 16.5 mm Hg,^21^ which is comparable to the 24 mm Hg decrease observed in dogs evaluated in our study. This observation is interesting in light of the fact that follow‐up PA pressures in the original study were obtained 1 hour to 112 days after initiation of sildenafil, in comparison to a standard 4‐week follow‐up in our study. In a prospective study of dogs with PH associated with chronic mitral valve disease,^24^ TRPG also decreased by 16 mm Hg after sildenafil treatment. Importantly, in that study, the estimated pressure gradient post‐sildenafil did not differ from that of control dogs treated with placebo. This finding could suggest that factors other than sildenafil contributed to the decrease in estimated PA pressure in that study, and a similar effect might be responsible for some of the results observed in our study. It is notable however that the use of additional medications for management of respiratory conditions did not impact results in the dogs of our study.

The decrease in estimated TRPG in dogs with respiratory‐associated PH after 1 month of sildenafil treatment could provide support for an active or vasoconstrictive component in the pulmonary vasculature associated with hypoxia, as has been suggested previously.^28^ Historically, dogs have been found to have a limited pulmonary vascular response to alveolar hypoxia,^29^ but many clinical studies have reported increases in estimated TRPG in dogs with chronic pulmonary diseases,^12^, ^13^, ^21^, ^22^, ^28^ despite a lack of blood gas data. Also, hypobaric hypoxia caused mild to moderate increases in systolic PA pressure (41‐55 mm Hg) in healthy exercising sled dogs,^30^ which suggests that global hypoxia triggers an increase in pulmonary vascular pressures even in normal dogs.

Not all studies have identified a decrease in TRPG after administration of sildenafil. A retrospective study of dogs with PH associated with cardiac or respiratory disease^22^ reported that estimated TRPG pre‐sildenafil did not differ from the result obtained 7‐521 days posttreatment. Average PAP in that study^22^ was in the moderate range (median, 62 mm Hg) compared to a majority of dogs having severe PH in the original study of sildenafil^21^ and in our study. Therefore, perhaps the variable response to sildenafil in these different studies was partly related to the severity of PH in affected dogs, but inconsistency in Doppler estimates of TRPG must be considered. It is also possible that the decrease in TRPG detected in our study was related to the dosage of sildenafil administered (4.2 mg/kg/day), which seems to be higher than that used in previous studies.^21^, ^22^, ^24^ This dosage was not only effective but also appeared to be safe because no adverse events were reported. Finally, genetic polymorphism in the phosphodiesterase 5A gene^31^ could contribute to variable response of the TRPG to sildenafil in different groups of dogs.

Based on clinical data and lack of echocardiographic evidence of heart disease, a respiratory‐associated cause of PH was assigned to all dogs in our study although, as indicated in Table 2, a specific respiratory condition could not be identified in 3 dogs with markedly abnormal radiographs, and 2 dogs were reclassified into groups 1 and 4 of the PH clinical classification scheme based on necropsy findings. The challenge in performing advanced diagnostic tests in dogs with respiratory disease and PH has been discussed in many other studies of PH.^12^, ^13^, ^14^, ^15^, ^20^, ^21^, ^22^ Although breed relationships, such as that of the West Highland White Terrier with pulmonary fibrosis, are widely used to confirm a diagnosis in clinical studies,^20^ these designations should always be interpreted cautiously.

In human medicine, the pathophysiologic events that result in PH remain obscure in most respiratory diseases. Hypoxic pulmonary vasoconstriction, alterations in release and activity of neurohormonal mediators, inflammation, and vascular remodeling likely contribute variably to the induction of PH across species and among individuals.^3^, ^4^, ^5^, ^6^ Brachycephalic breeds could be predisposed to pulmonary vascular remodeling because of intermittent hypoxemia, hypercoagulability, and systemic inflammation,^32^, ^33^, ^34^ Interestingly, 3 of 4 dogs that had worsening of QOL scores in our study were brachycephalic, with TBM and infection complicating the presentation in 2 dogs. Endothelin‐1, a potent vasoconstrictor and mitogen, has been implicated in the etiopathogenesis of idiopathic pulmonary fibrosis in the West Highland White Terrier, 35 which might partly explain the high incidence of PH in these dogs.^20^ More aggressive screening methods for dogs diagnosed with these conditions as well as with chronic bronchitis and TBM would allow determination of the overall prevalence of PH in these respiratory diseases, as has been done with mitral valve disease.^36^ These investigations also could help elucidate the contribution of different respiratory disorders to PH, and the variable response to sildenafil.

As described in earlier studies,^12^, ^21^, ^22^ syncope was a common finding in dogs in our study, with an overall occurrence of 64%. The percentage reported in our study was substantially higher than that previously described (32%)^22^ and much higher than the 7% reported in dogs with PH associated with myxomatous mitral valve disease (MMVD).^14^ It is possible that some of this difference is related to the severity of PH in different reports. In dogs with MMVD,^8^ <50% of dogs had PH that would be categorized in the moderate to severe range in comparison to all dogs in our study. Although dogs examined in our study had similar increases in TRPG regardless of the presence or absence of syncope, it is possible that mild PH is not sufficient to result in the pathophysiologic events that lead to syncope. It is also possible that challenges in estimating the TRPG make this variable too unreliable for statistical assessment, particularly given the relatively small number of dogs in our study. Finally, the lack of correlation of TRPG with the occurrence of syncope also might suggest that additional factors, such as the underlying respiratory condition, play a role in development of syncope, rather than the absolute increase in TRPG.

Previous studies on PH have reported improvement in QOL for dogs treated with sildenafil, but abbreviated or subjective clinical scores were employed. 22, 24 We elected to use a previously validated 17‐point QOL questionnaire that included multiple gradations of severity for assessment of dysfunction. Although the survey only has been validated for determining response to treatment in cardiac disease, review of the questions indicated substantial parallels to the clinical signs and treatment responses anticipated with respiratory diseases. Although use of this specific questionnaire does reflect a limitation of the study, the questionnaire appeared to perform well in dogs with respiratory conditions and identified an overall improvement in QOL in the dogs that survived the 1‐month treatment period.

Development of PH was reported to have relatively little prognostic relevance in a large‐scale study of dogs with mitral valve disease, with median survival of >15 months in affected dogs. 36 However, PH generally was less severe in those dogs and it was noted that a TRPG >55 mm Hg (which would be classified as moderate PH) was an independent predictor of poor outcome. All dogs in our study would fit into this category, yet in dogs that survived the 1‐month treatment period, initiation of sildenafil resulted in reasonable quantity and QOL in most dogs. Although TRPG did not correlate with survival in the original study assessing sildenafil responsiveness,^21^ our study found a significant but weak negative correlation. Addition of more dogs with mild PH might have strengthened that correlation, but it is equally possible that TRPG is just 1 of many factors that play a role in survival in dogs with PH. An early report on PH in dogs reflected a grim prognosis for survival with a median value of 3 days^12^ and the use of sildenafil in a subsequent study improved survival to 3 months.^21^ Despite the use of sildenafil in our study, 8 of 25 dogs (32%) did not survive through the first month of treatment. Clearly, overall survival of dogs with respiratory‐associated causes of PH in our study did not approach that found in dogs with PH associated with MMVD, but the 50% survival rate of 6 months for all dogs was considered acceptable, given the duration of clinical signs and the finding of moderate to severe PH, as well as the types and chronicity of respiratory diseases identified. In dogs that completed the 1‐month trial treatment period with sildenafil, 50% mortality approached 1 year, and additional large‐scale prospective studies of respiratory‐associated PH are needed to determine survival for different diseases.

Our study suffered from limitations common in studies involving clinical patients. Diagnoses in respiratory patients were not confirmed with airway sampling or histopathology in all cases, and subjective clinical variables were used to assign disease processes. Arterial blood gas evaluation rarely was performed in our study perhaps because of small patient size or concerns about stress to the patient, and definitive diagnoses sometimes were not sought because of concerns about invasive testing. Two dogs were clinically suspected to have interstitial fibrosis but on necropsy were determined to have pulmonary vascular and thromboembolic disorders. It is possible that other dogs in our study, as well as in other studies, were placed in the wrong classification for PH etiology. We did not assess caudal vena cava size^37^ or consider the presence of right‐sided heart failure in estimating PA pressure,^20^ which has been recommended as a possible method to more closely approximate PA pressure.^23^ Finally, echocardiography can overestimate or underestimate the pressure gradient as described earlier, and a recent study reported increased variance in echocardiographically derived PA pressure at higher pressures in comparison to direct catheterization studies.^23^ Median PA pressure in that study was 42 mm Hg, which is substantially lower than the pressures determined here, and increased variation in estimated PA pressure might be expected in our study. Also, the ability to obtain maximal TR jet varies with the operator and compliance of the animal,^22^ which despite best efforts potentially could hamper direct comparisons between measurements taken by different examiners on different days. Clearly, the difficulties in estimating PA pressure would impact all previous veterinary studies that have attempted to diagnose PH and assess response to sildenafil using echocardiographic measurements. Finally, almost one third of the dogs enrolled in our study died or were euthanized before reevaluation, which limited follow‐up assessment of TRPG and QOL scores.

Despite these limitations, our study confirmed improvement in QOL and an overall decrease in estimated PAP in dogs with respiratory‐associated causes of moderate to severe PH that were treated with sildenafil for 4 weeks. It also found a weak but significant effect of TRPG on survival, suggesting that the severity of PH as determined by Doppler echocardiography could be an important prognostic indicator in dogs with respiratory disease associated with PH, as it is in dogs with cardiac‐related PH. Our study also determined that PH can resolve if the underlying cause is identified and treated, as evidenced by the study dog that had resolution of PH and syncope with appropriate treatment for eosinophilic lung disease. With corticosteroid management and resolution of disease over 8‐10 months, the TRPG decreased to normal and sildenafil ultimately was discontinued. We found that survival of dogs with PH treated with sildenafil often exceeded 6 months, although substantial mortality was encountered shortly after the diagnosis and before response to treatment could be assessed. Importantly, severe clinical signs related to syncope and severely increased TRPG did not influence response to sildenafil. Finally, the utility of QOL scores should be further evaluated in patients with respiratory disease, with or without PH.

## CONFLICT OF INTEREST DECLARATION

Authors declare no conflict of interest.

## OFF‐LABEL ANTIMICROBIAL DECLARATION

Authors declare no off‐label use of antimicrobials.

## INSTITUTIONAL ANIMAL CARE AND USE COMMITTEE (IACUC) OR OTHER APPROVAL DECLARATION

Approval granted by University of California, Davis IACUC.

## HUMAN ETHICS APPROVAL DECLARATION

Authors declare human ethics approval was not needed for this study.
